# Influence of Statins on the Survival Outcomes of Patients with Diffuse Large B Cell Lymphoma: A Systematic Review and Meta-Analysis

**DOI:** 10.1155/2022/5618290

**Published:** 2022-07-30

**Authors:** Yanbing Li, Huijie Zhou, Liqun Zou

**Affiliations:** ^1^Department of Medical Oncology of Cancer Center, West China Hospital, Sichuan University, Chengdu 610041, China; ^2^Department of Oncology, Jiujiang No. 1 People's Hospital, Jiujiang 332000, China

## Abstract

**Background:**

Previous studies evaluating the influence of statins on the survival of patients with diffuse large B cell lymphoma (DLBCL) showed inconsistent results. This systematic review and meta-analysis was conducted to investigate whether statin use is correlated with the survival of DLBCL patients.

**Methods:**

Related cohort studies were obtained by searching PubMed, Embase, Cochrane's Library, and Web of Science databases. Study characteristics and outcome data were extracted independently by two authors. The random-effect model was used for meta-analysis, considering the possible influence of between-study heterogeneity.

**Results:**

Eight studies involving 9927 patients with DLBCL were included. Results did not show significant associations of statins with overall survival (OS, hazard ratio [HR]: 0.88, 95% confidence interval [CI]: 0.69∼1.11, *p*=0.27; *I*^2^ = 60%) or progression-free survival (PFS, HR: 0.92, 95% CI: 0.72∼1.17, *p*=0.49; *I*^2^ = 23%) in these patients. Subgroup analyses suggested that statin was be associated with survival of DLBCL patients from Asia (HR for OS: 1.19, 95% CI: 0.91∼1.56, *p*=0.19, *I*^2^ = 2%; HR for PFS: 1.13, 95% CI: 0.89∼1.44, *p*=0.33, *I*^2^ = 0%), but was associated with significantly improved survival of patients from Western countries (HR for OS: 0.73, 95% CI: 0.66∼0.81, *p* < 0.001, *I*^2^ = 0%; for PFS, HR: 0.72, 95% CI: 0.53∼0.96, *p*=0.03, *I*^2^ = 0%), which fully explained the heterogeneity (*p* for subgroup difference <0.05). Variables such as study design, patient age, and study quality were not shown to affect the findings.

**Conclusions:**

Overall, statins did not affect the survival of patients with DLBCL. However, statin use may be associated with an improved survival rate of DLBCL patients from Western countries.

## 1. Introduction

Diffuse large B cell lymphoma (DLBCL) accounts for up to 40% of total non-Hodgkin lymphomas (NHL) [1]. Currently, rituximab–cyclophosphamide, doxorubicin, vincristine, prednisone (R-CHOP) or R-CHOP–like chemoimmunotherapy has become the first-line chemotherapy for patients with DLBCL, with a curative rate of more than 60% [1–3]. However, about one third of the patients will still suffer from disease recurrence or relapse, leading to adverse events such as mortality [4, 5]. Statins are the most commonly prescribed lipid-lowering drugs, which function by inhibiting the 3-hydroxy-3-methylglutaryl coenzyme-A reductase, a key enzyme of cholesterol synthesis [6]. Besides, further investigations confirmed multiple pharmacological actions besides lipid-lowering, including anti-inflammation, proapoptosis, antiproliferation, immunomodulation, and antimetastasis, suggesting a possible role statin as an anticancer agent [7–9]. Indeed, statins have been shown to reduce the incidence of multiple solid tumors and hematological malignancies, including NHL [10, 11]. However, the influence of statin use on clinical outcomes of patients with DLBCL remains to be not fully determined. An early in vitro study showed that statins-induced cholesterol depletion may cause conformational changes of CD20 to escape detection by rituximab, thereby impairing the antilymphoma efficacy of rituximab in CD20^+^ B cell lymphomas [12]. Subsequent observational studies regarding the influence of statins on the prognosis of DLBCL patients showed inconsistent results [13–20]. An early study showed that statins may adversely affect the prognosis of patients with nongerminal center DLBCL [17], while a recent study suggested that concomitant use of statins may improve the survival of DLBCL patients who were treated with R-CHOP [20]. Accordingly, we performed a systematic review and meta-analysis to comprehensively evaluate the influence of statins on the survival of DLBCL patients.

## 2. Methods

The preferred reporting items for systematic reviews and meta-analyses (PRISMA) statement [21] was followed in this systematic review and meta-analysis. The methods of analyzing and reporting of the meta-analysis were consistent with the Cochrane's Handbook for Systematic Review and Meta-analysis [22].

### 2.1. Database Search

We systematically searched the four electronic databases, including PubMed, Embase, Cochrane's Library, and Web of Science using the combined keywords: (1) “3-hydroxy-3-methyl-glutarylCoA reductase inhibitor,” statin, statins, atorvastatin, rosuvastatin, fluvastatin, simvastatin, lovastatin, pitavastatin, or pravastatin; (2) lymphoma; and (3) survival, mortality, death, deaths, progression, recurrence, remission, or collapse. We only considered clinical studies, and no restriction was applied to the publication language. The citation lists of the related articles were also screened manually for possible relevant studies. The date of the final database search was January 12, 2022.

### 2.2. Study Inclusion

The PICOS criteria were followed during the determination of the inclusion criteria.

P (patients): adult patients with confirmed diagnosis of DLBCL; I (exposure): patients with statin use as defined by the original studies; C (control): patients without statin use as defined by the original studies; O (outcomes): relative risks for the incidence of overall survival (OS) and/or progression-free survival during follow-up durations.

S (study design): cohort studies, including the retrospective and prospective studies, published as full-length articles.

Reviews: studies including non-DLBCL patients, studies that did not evaluate the influence of statins, or studies that did not report the survival outcomes were removed.

### 2.3. Data Collection and Evaluation of Study Quality

Database search, data collection, and evaluation of study quality were separately performed by two independent authors. Discussion with the corresponding author was indicated to resolve the disagreements. Data regarding the study information, patient characteristics, details of statin use, follow-up durations, and survival outcomes were collected. Evaluation of study quality was achieved by the Newcastle-Ottawa Scale (NOS) [23]. This scale varies between 1 and 9 stars and assesses the quality of the cohort studies with three domains, including selection of the patients, comparability between patients with and without exposure, and strategies for the validation of the outcomes.

### 2.4. Statistical Methods

Hazard ratios (HR) and the 95% confidence interval (CI) were used to indicate the relative risk of survival outcomes between statin users and nonusers with DLBCL. If HRs with more than one model of multivariate regression analyses were reported, we collected the most adequately adjusted HR for subsequent analysis. With data of 95% CIs or *pP* values, standard errors (SEs) of HRs were calculated and HRs were logarithmically transformed to maintain a normal distribution [22]. Between-study heterogeneity was assessed by the Cochrane's *Q* test and estimation of the *I*^2^ statistic [24]. Typically, an *I*^2^ >50% was considered as the indicator of significant between-study heterogeneity. To minimize the influence of heterogeneity, we used the random-effect model to pool the HR data of each study in a conservative manner [22]. A series of subgroup analyses were conducted to reveal the influences of study characteristics on the associations according to variables such as study design, location, patient age, and study quality scores. The publication bias was assessed with the funnel plots and the Egger's regression test. We applied the RevMan (Version 5.1; Cochrane Collaboration, Oxford, UK) and Stata 12.0 software for the statistics of the meta-analysis.

## 3. Results

### 3.1. Identification of Related Studies

**Figure 1:**
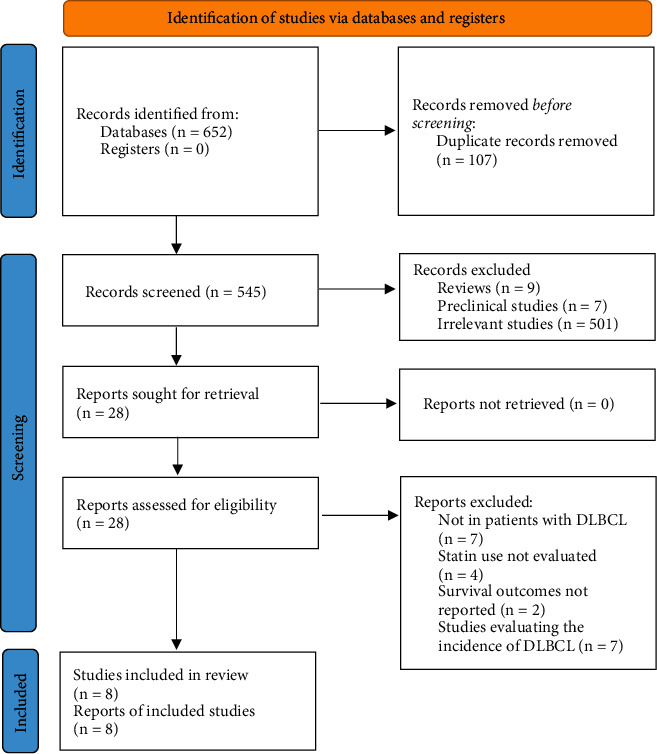
PRISMA diagram of literature search and study inclusion.

Figure 1 summarizes the process of literature search. In brief, 545 articles were retrieved in the initial database search after excluding the duplications. Then, 28 articles were considered to be potentially relevant after excluding 517 irrelevant articles in title and abstract screening. In the final step of full-text review, another 20 studies were excluded according to the reasons listed in Figure 1. Finally, eight cohort studies were identified and included in the meta-analysis [13–20].

### 3.2. Summary of Study Characteristics

Eight cohort studies [13–20] involving 9927 patients with DLBCL contributed to this meta-analysis. The characteristics of each study are displayed in Table 1. The studies were performed in Switzerland, Japan, the United States, Singapore, Korea, Canada, and Sweden. Two of them were prospective [14, 19], while the others were retrospective. All of the studies included patients with DLBCL. In six studies, R-CHOP or R-CHOP–like chemotherapy was the primary treatment [13–17, 20], while the other two studies did not specify the details of treatments [18, 19]. Statin use was defined as concurrent use via medical charts validation for six studies [13–17, 20], while it was defined as previous use before enrollment in two studies [18, 19]. Accordingly, 2847 (28.7%) patients were statin users. The mean follow-up durations were 12–77 months, and confounding variables such as age, sex, stage of lymphoma, and performance status were controlled in the multivariate analyses. The NOS for the studies varied between six and nine, which suggested good quality of the included cohort studies (Table 2).

### 3.3. Statins and OS  of DLBCL

**Figure 2:**
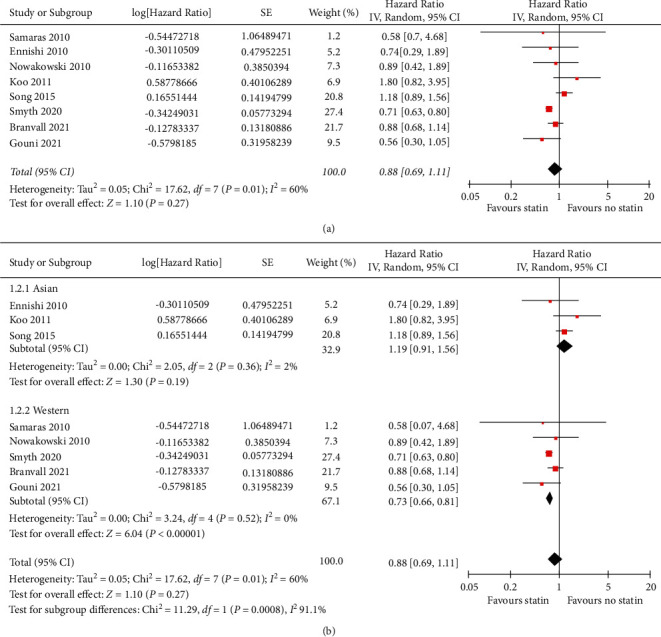
Forest plots for the meta-analysis of the association between statin use and OS of DLBCL. (a) Overall meta-analysis; and(b) subgroup analysis according to the country of the study.

Pooled results of eight cohorts demonstrated that statins were not significantly associated with OS of patients with DLBCL (HR: 0.88, 95% CI: 0.69∼1.11, *p*=0.27; Figure 2(a)) with significant heterogeneity (*p* for Cochrane's *Q* test = 0.01, *I*^2^ = 60%). Sensitivity analysis was limited to six studies [13–17,20] which applied R-CHOP or R-CHOP–like chemotherapy as the primary treatment and showed that concurrent use of statin did not significantly affect OS of these patients (HR: 0.97, 95% CI: 0.70∼1.37, *p*=0.88, *I*^2^ = 32%). Subgroup analysis failed to show that statin use was associated with OS of patients from Asia (HR: 1.19, 95% CI: 0.91∼1.56, *p*=0.19, *I*^2^ = 2%), but statins were found to be associated with a significantly improved OS of patients from Western countries (HR: 0.73, 95% CI: 0.66∼0.81, *p* < 0.001, *I*^2^ = 0%; Figure 2(b) and Table 3). The between-subgroup difference was statistically significant (*p* < 0.001), which fully explained the heterogeneity of the meta-analysis. Subgroup analysis according to other variables did not show a significant association (Table 3).

### 3.4. Statins and PFS of DLBCL

**Figure 3:**
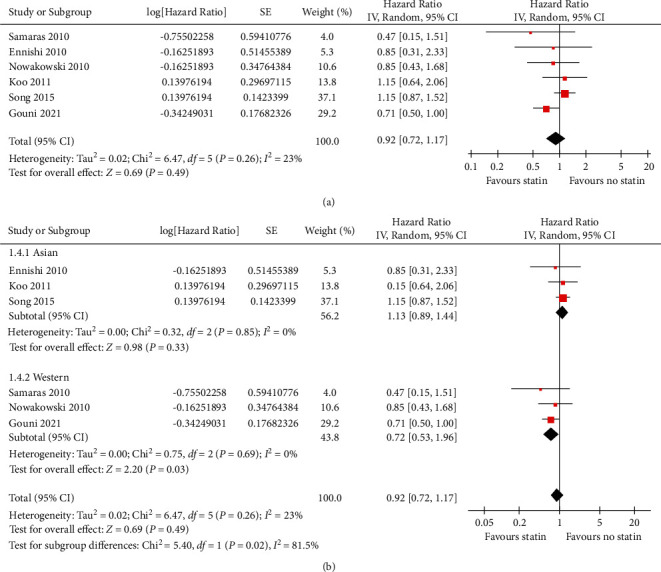
Forest plots for the meta-analysis of the association between statin use and PFS of DLBCL. (a) Overall meta-analysis; and(b) subgroup analysis according to the country of the study.

Pooled results of six studies [13–17, 20], all using R-CHOP or R-CHOP–like chemotherapy as the primary treatment, showed that concurrent statin use was not associated with PFS of DLBCL patients (HR: 0.92, 95% CI: 0.72∼1.17, *p*=0.49; I^2^ = 23%; Figure 3(a)). Subgroup analysis showed statin use did not significantly affect PFS of patients from Asia (HR: 1.13, 95% CI: 0.89∼1.44, *p*=0.33, *I*^2^ = 0%), but it was associated with an improved PFS in patients from Western countries (HR: 0.72, 95% CI: 0.53∼0.96, *p*=0.03, *I*^2^ = 0%; Figure 3(b) and Table 3). The between-subgroup difference was significant (*p*=0.02), which fully explained the between-study heterogeneity. Subgroup analysis according to other study characteristics failed to show a significant association between statins and PFS in patients with DLBCL (Table 3).

### 3.5. Publication Bias

**Figure 4:**
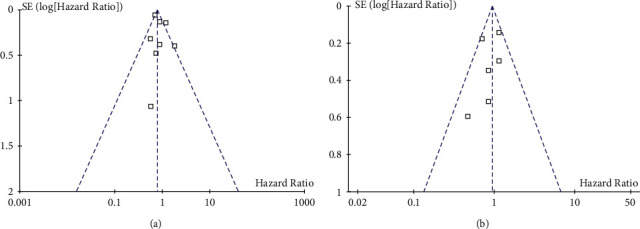
Funnel plots for the meta-analyses; (a) funnel plots for the association between statin use and OS of DLBCL; and (b) funnel plots for the association between statin use and PFS of DLBCL.

Funnel plots for the outcomes of OS and PFS were symmetrical on visual examination (Figures 4(a) and 4(b)), suggesting low risk of publication biases, which were further confirmed by the results of Egger's regression tests (*p*=0.32 and 0.29. respectively).

## 4. Discussion

In this meta-analysis, by pooling the results of eight relevant cohort studies, the results showed that overall, statin use did not seem to significantly affect outcomes of OS or PFS in patients with DLBCL, even limited to studies including patients treated with R-CHOP or R-CHOP-like chemoimmunotherapy. Moreover, subgroup analysis indicated that concurrent statin use may be associated with improved OS and PFS in patients with DLBCL from Western countries, but not in patients from Asia. Taken together, these findings indicate that statin use does not impair the survival of DLBCL but may improve the survival of DLBCL patients from Western countries.

To the best of our knowledge, this is the first meta-analysis evaluating the prognostic influence of statins in patients with DLBCL. An early meta-analysis including five cohort studies showed that statin use did not affect OS in patients with NHL or chronic lymphocytic leukemia (CLL) [11]. However, mixed patients with DLBCL, follicular lymphoma (FL), and CLL were included, and the differences in the course of the diseases and treatment regimens made the results difficult to interpret [11]. Our study included eight up-to-date cohort studies involving patients with DLBCL only, and showed that statin use did not affect the survival outcome in these patients. Sensitivity analysis limited to studies with concomitant statins in patients treated with R-CHOP or R-CHOP-like chemoimmunotherapy showed consistent results. A previous preclinical study showed that statins may compromise the antilymphoma activity of rituximab via inducing conformational changes of CD20 [12]. The results of our meta-analysis confirmed that the findings of the preclinical study may have no significant clinical implications. One possible explanation is that the possible interference of concomitant statins on the efficacy of rituximab may be offset by other possible anticancer actions of statins. For example, a recent preclinical study in DLBCL cell lines showed that lovastatin improved doxorubicin sensitivity, an important component of the R-CHOP regimen [20]. Besides, accumulating evidence in studies of patients with other diseases that were treated with rituximab also showed that concomitant statins did not significantly affect the clinical outcomes, such as in those with FL [25], CLL [26], or rheumatoid arthritis [27]. Collectively, the findings of our meta-analysis showed that statin use does not impair the survival of DLBCL, even with concomitant statin use in patients treated with rituximab-related chemoimmunotherapy.

Interestingly, results of the subgroup analysis showed that the countries of the study may affect the association between statin use and survival of DLBCL, which fully explains the source of the between-study heterogeneity. Particularly, we found that statin use may be associated with improved survival in DLBCL patients from Western countries but not in patients from Asia. These findings may suggest a possible racial difference for the influence of statin use on outcomes in DLBCL patients. The potential racial difference for the survival of DLBCL was also observed in a recent study, which showed that Hispanics/Latinos had improved survival compared to non-Hispanics [28]. Currently, the potential mechanisms underlying these findings are not known. One explanation is that the possible interfering of satin on the antilymphoma efficacy of rituximab was mediated on cholesterol depletion [12], which may be affected by the level of serum cholesterol. Patients from Western countries generally have higher serum cholesterol levels than patients from Asia [29], which makes them less vulnerable to the interference of statins. Consistently, a previous study showed that low serum cholesterol levels are a predictor of poor prognosis in DLBCL patients [30]. Studies are needed in the future for the validation of these findings and to reveal the underlying mechanisms.

Our study has limitations. Firstly, the number of the included cohorts was small, and the sample sizes were limited. We could not determine the possible influences of some patient or study characteristics on the outcomes, such as gender of the patients, comorbidities, and regimens of chemotherapy. Besides, influences of type, dose, and duration of statin use on the survival of patients with DLBCL remain to be determined. We could not evaluate the possible influences of these factors because related data were rarely reported among the included original studies. In addition, the different countries of the study may not accurately reflect the different races of the patients. Future large-scale prospective cohort studies may be considered for validation. Finally, this meta-analysis is based on observational studies, the results of which may be affected by selection bias, recall bias, and residual confounding factors. Clinical studies may be considered to evaluate the possible survival benefit of statins in DLBCL patients from Western countries.

## 5. Conclusions

In conclusion, results of the meta-analysis showed that statin use does not impair the survival of DLBCL, even with concomitant statins in DLBCL patients treated with R-CHOP or R-CHOP-like chemoimmunotherapy. Moreover, concomitant statins may improve the survival of DLBCL patients from Western countries. Clinical studies are needed to validate these findings, and future studies are warranted to clarify the possible underlying mechanisms.

## Figures and Tables

**Table 1:** Study characteristics.

Study	Design	Location	No. of patients	Mean age	Men	Therapy	Stage	Definition of statin use	Number of patients with statin use	Follow-up durations	Variables controlled	Outcomes reported
				years	%					months		

Samaras 2010	R	Switzerland	145	57.3	56.6	R-CHOP as first line therapy	I–IV	Concurrent use of statin as evidenced by medical chart	21	24	Age, sex, stage, and IPI	OS and PFS

Ennishi 2010	R	Japan	256	64.5	55.9	R-CHOP as first line therapy	I–IV	Concurrent use of statin for at least two months as evidenced by medical chart	35	32	Age, sex, cholesterol profile, and IPI	OS and PFS

Nowakowski 2010	P	The US	228	NR	56	R-CHOP or R-CHOP–like chemotherapy	I–IV	Concurrent use of statin as evidenced by medical chart	39	47	Age, sex, stage, and IPI	OS and PFS

Koo 2011	R	Singapore	213	59.1	50.7	R-CHOP or R-CHOP–like chemotherapy	I–IV	Concurrent use of statin as evidenced by medical chart	47	17	Age, sex, stage, performance status, serum LDH and chemotherapy regimens	OS and PFS

Song 2015	R	Korea	409	61	57.2	R-CHOP	I–IV	Concurrent use of statin as evidenced by medical chart	146	39	Age, sex, stage, and performance status	OS and PFS

Smyth 2020	R	Canada	4275	75	51	NR	I–IV	Previous use of statin within 1 year before enrollment	1607	12	Age, sex, and income level	OS

Branvall 2021	P	Sweden	4130	69	57.2	NR	I–IV	Previous use of satin before the enrollment	873	25	Age, sex, year of diagnosis, education, and concomitant medications	OS

Gouni 2021	R	The US	271	NR	49	R-CHOP	III–IV	Concurrent use of statin as evidenced by medical chart	79	77	Age, sex, stage, and performance status	OS and PFS

R, retrospective; P, prospective; US, United States; R-CHOP, rituximab–cyclophosphamide, doxorubicin, vincristine, prednisone; IPI, international prognostic index; LDH, lactate dehydrogenase; OS, overall survival; PFS, progression-free survival.

**Table 2:** Details of study quality evaluation.

Study	Representativeness of the exposed cohort	Selection of the nonexposed cohort	Ascertainment of exposure	Outcome not present at baseline	Control for age and sex	Control for other confounding factors	Assessment of outcome	Enough long follow-up duration	Adequacy of follow-up of cohorts	Total
Samaras 2010	0	1	1	1	1	1	1	1	1	8
Ennishi 2010	0	1	1	1	1	1	1	1	1	8
Nowakowski 2010	1	1	1	1	1	1	1	1	1	9
Koo 2011	0	1	1	1	1	1	1	0	1	7
Song 2015	0	1	1	1	1	1	1	1	1	8
Smyth 2020	1	1	0	1	1	0	1	0	1	6
Branvall 2021	1	1	0	1	1	0	1	1	1	7
Gouni 2021	0	1	1	1	1	1	1	1	1	8

**Table 3:** Subgroup analyses for the outcomes of BW and BMI.

Characteristics	OS					PFS				
	No. of studies	HR (95% CI)	*I* ^2^	P1	P2	No. of studies	HR (95% CI)	*I* ^2^	P1	P2
Study design										
Prospective	2	0.88 (0.69, 1.12)	0%	0.31		1	0.85 (0.43, 1.68)	—	0.64	
Retrospective	6	0.88 (0.62, 1.25)	70%	0.47	1.00	5	0.92 (0.69, 1.23)	37%	0.54	0.84

Country										
Asian	3	1.19 (0.91, 1.56)	2%	0.19		3	1.13 (0.89, 1.44)	0%	0.33	
Western	5	0.73 (0.66, 0.81)	0%	<0.001	<0.001	3	0.72 (0.53, 0.96)	0%	0.03	0.02

Mean age										
≤60 years	2	1.56 (0.75, 3.26)	0%	0.23		2	0.85 (0.37, 1.95)	45%	0.71	
>60 years	4	0.87 (0.66, 1.15)	75%	0.33	0.14	3	0.91 (0.62, 1.33)	56%	0.62	0.89

Quality scores										
6–7	3	0.86 (0.63, 1.18)	72%	0.35		1	1.15 (0.64, 2.06)	—	0.64	
8–9	5	0.90 (0.64, 1.26)	26%	0.54	0.86	5	0.87 (0.66, 1.16)	33%	0.36	0.41

OS, overall survival; PFS, progression-free survival; HR, hazard ratio; CI, confidence interval; P1, *p* values for subgroup effect; P2, *p* values for subgroup difference.

## Data Availability

The data used to support the findings of this study are available from the corresponding author upon request.
